# Associations between Parents’ Body Weight/Shape Comments and Disordered Eating Amongst Adolescents over Time—A Longitudinal Study

**DOI:** 10.3390/nu15061419

**Published:** 2023-03-15

**Authors:** Lucy M. Dahill, Phillipa Hay, Natalie M. V. Morrison, Stephen Touyz, Deborah Mitchison, Kay Bussey, Haider Mannan

**Affiliations:** 1 Translational Health Research Institute, School of Medicine, Western Sydney University, Sydney, NSW 2051, Australia; p.hay@westernsydney.edu.au (P.H.); n.morrison@westernsydney.edu.au (N.M.V.M.); deborah.mitchison@westernsydney.edu.au (D.M.); h.mannan@westernsydney.edu.au (H.M.); 2 South West Sydney Local Health District, Camden and Campbelltown Hospitals, Campbelltown, NSW 2560, Australia; 3 School of Psychology and Inside Out Institute, University of Sydney, Sydney, NSW 2006, Australia; stephen.touyz@sydney.edu.au; 4 Sydney Local Health District, Camperdown, NSW 2050, Australia; 5 Centre for Emotional Health, Macquarie University, North Ryde, NSW 2109, Australia; kay.bussey@mq.edu.au

**Keywords:** communication, parents, adolescent, teasing, mental health, eating disorder

## Abstract

Parents are key influencers of adolescents’ attitudes on weight, shape, and eating, and make more positive than negative comments, with negative comments most impactful. This study examined prospective unique associations of parental positive and negative comments in a community sample of adolescents with paediatric psychosocial quality of life (PED-QoL), Eating Disorder Weight/Shape Cognitions (EDEQ-WS), BMI percentile, and Psychological Distress (K10) scales. Data were from 2056 adolescents from the EveryBODY study cohort. Multiple regressions were conducted for the impacts of parental positive and negative comments on four dependent variables at one year after controlling for their stage of adolescence (early, middle, late). Multiple imputation and bootstrapping were used for handling missing data and violations of normality. Results indicated that positive maternal comments on eating were associated with increased EDCs and better quality of life at one year. Paternal positive weight shape comments were associated with a decrease in psychological distress, but positive eating comments saw a decrease in quality of life. Findings highlight the nuances of parental comments and how these are perceived and interpreted, and could alert health care workers and family practitioners who have weight, shape, and eating conversations to be aware of the potential influence of their communication.

## 1. Introduction

Parents have been found to be key influences during childhood setting standards for behaviour that continue into adult life [1,2,3]. Whilst parental influence can diminish in middle adolescence [4,5,6], it has been found to have an enduring influence on physical and mental health outcomes with adolescents, particularly for eating disorder cognitions (EDCs), setting longer term foundations of communication and behaviours relating to eating, weight, and shape [5,7,8]. Adolescence is also a key life-stage for the onset of eating disorder risk factors, including body dissatisfaction, low self-esteem, and weight shape concerns [9,10]. Furthermore, it is a life-stage associated with biological, psychological, and emotional change, with complex challenges involving maturation and social emotional development that can impact quality of life and influence the onset of eating disorders [11,12]. Parental comments and weight-talk between parents and adolescents requires further exploration for their health-related and psychosocial outcomes [13].

Parents’ concern for their child’s wellbeing and challenges with their growing independence can result in conflictual communication and miscommunications around weight, shape, and eating [5,8,14,15]. The influence of communication can be direct, such as verbal and targeted (e.g., parent to child), or indirect, such as non-verbal (e.g., eye movements) or when speaking about someone else (e.g., “they look great with a little less weight on their hips”) [13,16,17]. Mothers have been found to comment more frequently, particularly to daughters about their bodies and their eating [5,7,18,19,20,21,22], and maternal delivered content has been more attuned to thin-idealisation, while alternations, such as muscle-ideation, are more likely to be reported in father–son communications [19,23].

### 1.1. Positive and Negative Comments

Research has investigated the prevalence of positive and negative eating and weight/shape-related comments [5,7], the importance and associations of these comments with mothers and fathers [5,20,24], associations between weight talk exposure, psychological distress, and unhealthy eating and/or weight control behaviours [13,15,17], maternal encouragement to diet with boys and girls [25], and the perception and nuances of such communication [5].

In previous research, we [7], examined the prevalence of positive and negative comments to sons and daughters in this adolescent sample. Adolescents reported frequent positive comments more commonly from mothers (78%) than fathers (51%) on weight and shape and on eating (mothers 70% versus fathers 53%). Furthermore, daughters, compared to sons, reported more positive (mothers 85% versus fathers 71%) and negative (mothers 40% versus fathers 33%) comments on weight/shape, and more negative eating comments from their mothers (mothers 66% versus fathers 57%). Fathers were reported to comment more negatively on sons’ weight/shape than on daughters (mothers 25% versus fathers 32%). Further, for all perceived parental negative weight shape comments and maternal negative eating comments, adolescent stage and biological sex were significantly associated with EDCs and psychological distress [24].

Negative comments can take many forms. For example, a well-recognised form is teasing. Such negative comments may be presented as a form of humour and present a form of humour at a developmental stage of life where markers for understanding such complex forms of communication have not yet developed [26]. Of note, when considering the content of the comments, fathers have been found to be more likely to “tease” both their sons and daughters compared to mothers [27,28]. Furthermore, studies have shown that differing “modes of influence”, such as parents, peers, and other influences can act in concert or counteractively [23] with a cumulation of comments (positive or negative) from parents being associated with more negative outcomes and a lower quality of life than from either parent alone [29].

Longitudinal studies have also considered the long-term effects of negative comments, such as those delivered through weight-based teasing [30,31], the pertinence of age [6], associations of parental comments with EDCs including psychological distress [32], parental teasing and eating disorder risk factors [28], parents as influencing factors [6,33], and considered if the gender of the source of the weight teasing (parents or peers) influenced the effect of appearance and weight esteem [34]. Findings suggest this form of negative communication in families during adolescence leads to a greater risk of unhealthy weight control behaviours (UWCBs) in early adulthood [28,31,32,34], and effects have been shown to vary by gender. Puhl (2017) [31] found women to be more adversely affected by negative maternal comments 15 years later, and although Valois et al., (2019) [34] did not find any difference in effect dependent on the gender of the parent performing the appearance teasing, they did suggest that the teaser’s gender should be considered in weight-teasing prevention strategies. A systematic review of population-based studies which included two longitudinal studies considered psychosocial quality of life to be associated with disordered eating behaviour in adolescents [35]. Whilst there are multiple factors that influence quality of life in adolescents, it is imperative that we further consider the long-term effects of negative comments which could adversely influence adolescent psychological and physical health. To our knowledge, there is no other paper that examines comprehensively this topic across both genders in both parents and children, i.e., positive and negative comments about weight shape and eating from both mothers and fathers during adolescence.

### 1.2. Research Question

This study aims to explore the associations of mothers’ and fathers’ positive and negative comments on sons and daughters’ psychosocial quality of life, eating disorder cognitions (EDCs), BMI percentile, and psychological distress over time, thereby extending our cross-sectional research [24]. Thus, we will provide more empirical evidence and understanding to inform parents and clinicians working with families in this area around appropriate communication about weight and shape concern.

Based on the above previous research, we hypothesised the following:

i. Perceived positive parental comments on shape/weight would be significantly associated at the one-year follow-up with lower psychological distress, BMI percentile and eating disorder cognitions for daughters;
ii. All perceived negative parental comments on shape/weight or eating would be significantly associated at the one-year follow-up with greater psychological distress, BMI percentile, and EDCs for both sons and daughters;
iii. Perceived positive eating comments from mothers and fathers would be significantly associated at the one-year follow-up with reduced psychological distress, BMI percentile, and EDCs.
iv. Associations of all perceived comments from mothers and fathers and associations with quality of life are exploratory, but we anticipated that perceived negative comments may be associated with a lower quality of life and that perceived positive comments with a better quality of life at one-year follow-up.

## 2. Methods

Data for this study were drawn from a subset of the EveryBODY study, a longitudinal investigation of body image concerns and eating disorders among Australian adolescents. At the time of data collection there had been three data collection points (waves). Included participants captured in both wave 2 (T1) and the one-year follow-up at wave 3 (T2) were from four private and four public schools in New South Wales. The sub-sample in this study were those participants who had indicated they had a mother or father in their life and who completed questions related to parental comments at T1 of the survey. The final sample for T1 (*n* = 2204) consisted of 46% boys, and less than 1% reported a non-binary gender or did not respond to this item; participants were aged between 11 and 18 years, with a mean age 14.84 years (*SE* = 0.58). A variable ‘stage’ was created to indicate early, middle, and late adolescence. Early was classified as grades 7 and 8 (40.9%) which represented the first two years of high school in Australia, usually around 12–14 years; grades 9 and 10 (42.3%) indicated middle adolescence, and grades 11 and 12 (16.8%) were used to indicate late adolescence. These ‘stages’ have been used in previous research [7,36,37]. There were 2056 respondents at the one-year follow-up (T2), and the attrition in the sample was 7% and consisted of 46% boys; less than 1% reported a non-binary gender or did not respond to this item.

### 2.1. Measures

#### 2.1.1. Specific Parental Comment Questions

Maternal and paternal comments on weight/shape and on eating were measured using purpose-designed items by the authoring team and described in previous research [7]. Participants answered an initial question, “Is your mother in your life?” (or mother figure). Responses were binary (yes/no). Those who gave an affirmative response were asked a series of two questions regarding the frequency of maternal comments on (i) weight and shape and (ii) eating. Responses were rated on a 5-point scale: “never” (1), “rarely” (2), “sometimes” (3), “often” (4), and “all of the time” (5). The same questions were asked regarding comments from fathers or father figures. There was a maximum of eight questions in total, because if they answered “no” to either the mother or father question, that reduced the number to below eight. These were only collected at T1. The full list of questions is in Supplementary File S1.

#### 2.1.2. Eating Disorder Cognitions

Twelve items from the Weight Concern and Shape Concern subscales of the Eating Disorder Examination Questionnaire (EDEQ-WS) were used to assess eating disorder psychopathology, referred to as eating disorder cognitions (EDCs) in this study. Participants are asked questions that related to weight/shape concerns over the past 4 weeks (28 days), such as “how dissatisfied have you been with your shape?” Responses were measured with a Likert scale (0 = Not at all through to 6 = Markedly). A mean of the 12 items was used to compute a global score, ranging from 0 to 6, with higher scores conferring greater dissatisfaction. The combined EDEQ-WS score has been used to define overall body image disturbance in the diagnosis of anorexia nervosa [38]. McDonald’s omega estimates for the reliability for the combined weight and shape concern subscale in the present study was 0.96 and 0.94 for girls and boys, respectively [36]. A higher EDEQ-WS score means a higher risk of EDCs. These were collected at T1 and T2.

#### 2.1.3. Psychological Distress

The K-10 Psychological Distress Scale [39] was used to ask about the frequency of anxiety and depressive symptoms in the past 4 weeks. Participants completed 10 items on how often they experienced specific feelings (e.g., “Tired out for no good reason”) in the last 28 days (4 weeks) on a 5-point Likert scale (1 = None of the time through to 5 = All of the time). Scores range from 10 to 50, with higher scores indicating higher levels of distress. This scale has demonstrated high clinical utility in predicting clinically significant levels of distress in adolescent populations [39,40,41]. McDonald’s omega for the K-10 in the present study was 0.94 and 0.93 for girls and boys, respectively [36]. These were collected at T1 and T2.

#### 2.1.4. Quality of Life

Quality of life was measured using the psychosocial functioning subscale of the Paediatric Quality of Life scale (PED-QoL) [42,43]. Participants were asked to rate on a 5-point Likert scale (1 = Never through to 5 = Always) how true a series of statements were of them in the past 4 weeks. Scores were combined, reversed, and transformed on a 0–100 scale, with a higher score indicated higher functioning i.e., a better psychosocial quality of life. The psychosocial functioning subscale is a combination of the emotional and social functioning scales. In previous studies with adolescents, the psychosocial functioning subscale has shown good reliability and validity [43]. McDonald’s omega for the PED-QoL in the present study was 0.90 and 0.91 for girls and boys, respectively [36]. These were collected at T1 and T2.

#### 2.1.5. Body Mass Index Percentile (BMI%ile)

Height and weight were derived from self-report. Body mass index (BMI) (Kg/m^2^) was calculated and then converted to sex- and age-adjusted BMI percentiles (BMI%iles). These were derived according to the Centers for Disease Control and Prevention guidelines (2017). These were collected at T1 and T2.

### 2.2. Data Analysis

Intention to treat analyses were used. Multiple regressions were conducted for four dependent variables, namely psychosocial PED-QoL, K10, EDEQ-WS subscale, and BMI percentile at Time 2 (wave 3) on parental positive and negative comments at Time 1 (wave 2), after controlling for gender and baseline adolescent stage. Multiple imputation and bootstrapping were used for handling missing data and violations of normality, respectively.

For fitting multiple linear regressions, all the dependent variables at T2, namely K10, BMI%ile, EDEQ-WS combined, and psychosocial PED-QoL were found to be not normally distributed, thus, violating the assumption. Consequently, 1000 bootstrap samples were generated to correctly determine the standard errors and 95% confidence intervals for the regression coefficients in multiple linear regression. To account for missing data, the multiple imputation method of 10 imputations was used. The multivariate normal imputation (MVNI) with MCMC algorithm was used for this purpose. All analyses were performed using SAS 9.4 (SAS Institute, Cary, NC, USA, 2013).

Sex and baseline stage were not both controlled for throughout the analyses. With the imputed data for T2, the sample size used for multiple linear regression of the relevant T2 outcome was 2056 after controlling for the baseline stage only. However, if we had also controlled for sex, the sample size used for multiple linear regression of the T2 outcome would have been only 1639, hence, losing statistical power. The results presented did not control for sex to avoid the loss of statistical power when the dependent variables were psychological distress and BMI percentile as sensitivity analysis showed that sex was not a confounder for the relationship between comment variables and these outcomes. However, when the dependent variables were psychosocial PED-QoL and EDEQ-WS combined, sex was found to be a confounder and, thus, was appropriately controlled for in the analysis alongside baseline stage.

## 3. Results

The prevalence of comments has been previously reported [7] and further illustrated in the introduction for this paper.

### 3.1. Psychological Distress

After controlling for baseline stage, both more frequent positive (RE: 0.99; 95% CI: 0.67–1.30; *p* < 0.000) and more frequent negative (RE: 0.45; 95% CI: 0.012–0.90 *p* < 0.05) maternal weight shape comments at baseline were significantly related to higher psychological distress one year later. On the other hand, greater frequency of positive paternal weight/shape comments were found to be associated with lower psychological distress after one year (RE: −0.41; 95% CI: −0.80–−0.00 *p* < 0.05). These results are reported in Table 1. When exploring just daughters (see Table 2), only more frequent maternal positive weight/shape comments were associated with greater psychological distress (RE: 0.55; 95% CI: 0.04–1.05 *p* < 0.05). All other effects on psychological distress for daughters were non-significant. When exploring just sons, none of the comment variables were significant (see Table 3).

### 3.2. BMI Percentile

There were no significant comment variables when exploring parental comments to all adolescents (see Table 1); however, when exploring gendered dyads, mothers’ positive comments to sons on eating were significantly related to higher BMI at one-year follow-up (RE: 2.49; 95% CI: 0.57–4.41 *p* < 0.05) (see Table 3). For daughters, there were no significant comment variables (see Table 2).

### 3.3. Eating Disorder Cognitions (EDCs, EDEQ-WS)

Paternal negative eating (RE: 0.02; 95% CI: 0.02–0.12 *p* < 0.05) and maternal positive eating (RE: 0.06; 95% CI: 0.01–0.11 *p* < 0.01) comments were found to be significantly associated with greater increases in EDCs at 1 year (see Table 1). When looking for gendered effects, only paternal negative eating comments to daughters was significant and associated with greater increases in EDCs (RE: 0.15; 95% CI: 0.01–0.28 *p* < 0.05). This is reported in Table 2.

### 3.4. Paediatric Quality of Life (PED-QoL)

All maternal comments, except for maternal negative eating comments, were significant for better quality of life (see Table 1). All paternal comments were significant with mixed associations. Whilst paternal positive weight/shape (RE: 2.0; 95% CI: 1.37–2.62 *p* < 0.000) and negative eating (RE: 0.74; 95% CI: 0.05–1.44 *p* < 0.05) comments were significant for better quality of life, paternal negative weight/shape (RE: −0.92; 95% CI: −1.72–−0.07 *p* < 0.05) and positive eating (RE: −1.56; 95% CI: −2.12–−0.95 *p* < 0.01) comments were associated with a decrease in quality of life. For gendered effects, only maternal positive eating comments to sons were significant and associated with an increased quality of life (RE: 1.87; 95% CI: 0.28–3.45 *p* < 0.05). This finding is reported in Table 3.

## 4. Discussion

This study followed a diverse range of adolescents to examine longitudinal associations between perceived positive and negative parental comments on weight, shape, and eating and quality of life, BMI percentile, psychological distress, and EDCs one year later. It considered if this relationship was influenced by stage of adolescence and sex. Findings illustrated the complexity of how words are perceived by adolescents and the adverse outcomes that are associated with both positive and negative comments on weight shape and eating from both mothers and fathers to sons and daughters. Hypothesis 1 was partially supported, as only fathers’ positive comments on weight and shape were significantly associated at one-year follow-up with lower psychological distress. Hypothesis 2 was also partially supported, as only maternal negative weight and shape comments were significantly associated with greater psychological distress. Hypothesis 3 was not supported, as perceived positive eating comments from mothers or fathers were not significantly associated at one-year with reduced psychological distress. Although hypothesis 4, that associations of all comments from mothers and fathers and associations with PED-QoL were exploratory, the anticipated correlation between positive comments being associated with higher score for PED-QoL were not consistently supported with fathers’ positive eating and negative weight/shape comments, which were found to be associated with a decrease in quality of life. The complex nature of what contributes to quality of life suggests that our results warrant further investigation.

### 4.1. Maternal Comments

The present study found that more frequent comments from mothers about weight/shape and eating was a significantly predictive factor of EDCs and psychological distress, and that gendered effects were more pronounced with daughters [5,8,20,29]. However, this was not the case for quality of life, where mothers’ communications appeared to become protective and associated with increased quality of life. This could suggest that mothers are potentially more attuned and willing to have those conversations, which is confirmed by prevalence data on maternal comments [5,19,20,22], and, therefore, such comments can create an environment which add to a positive quality of life, even if the comments are not always perceived as positive. However, there are many other contributing factors, such as parental involvement in the home and other family or community influences, which could have contributed to our findings. The statistically significant association between wave 1 and wave 2 EDEQ-WS, K-10, psychosocial PED-QoL, and BMI percentile in all regressions suggests they naturally have an influence on later measures of emotional and psychological wellbeing, and may also be a mediating risk factor for these associations later in life, which could be explored in subsequent research. Of note, maternal positive eating comments to sons were associated with an increase in BMI which could contribute to the fear of talking positively to sons about their eating out of concern that it will encourage them to eat more. This is an area that could be further explored in subsequent mixed-method research exploring parental concerns about the consequences of healthy eating conversations [5,13].

### 4.2. Paternal Comments

In this and other studies, fathers’ comments had an anticipated association, with positive comments on weight and shape being significantly associated with lower psychological distress and EDCs at one-year for daughters [5,19,23]. However, positive comments did not have the same significance. Of note, positive paternal eating comments were not protective of PED-QoL, with or without controlling for baseline stage. Whilst research is sparse, fathers have been found to be more likely to tease or to use humour and sarcasm at a time where their adolescents do not yet have the cognitive ability to understand such complex forms of communication, which could, therefore, encourage literal and detrimental interpretation [23,26,28,44]. Their comments, although less prevalent than mothers, can have significant associations with EDCs and psychological distress [13,27,45]. The findings ask us to explore whether one parents’ comments are more influential, or if the differing modes of influence are acting in concert, leading to the cumulation of comments providing fertile soil for more sensitivity to comments from one parent or another [5,20,23,29,33]. This could extend to the other influences on an adolescents’ physical and psychological wellbeing, peers, siblings, the media, what is said around them, and how their parents talk about their own weight, shape and eating [3,15,17,28,33,34].

### 4.3. Psychosocial Quality of Life

Psychosocial quality of life in adolescence has been explored as influencing the onset of eating disorders [11], yet the paucity of research specific to mothers and fathers’ perceived positive and negative weight, shape, and eating comments to adolescents and associations with psychosocial quality of life offers an opportunity to consider the nuance of gendered and didactic communication in families, the gender of the parent having more of these conversations, and how much value we place on those conversations, as called for in a recent systematic review [13]. Our psychosocial quality of life findings highlight the complexity of parental communication and the nuanced perception of positive and negative comments within the context of other influences in adolescents’ lives. These counter-intuitive results may be interpreted to illustrate that there is no clear rule to share with parents and health care professionals about the valence of words to use when talking to adolescents about weight, shape, and eating, as there are often complex contributing factors to how words are perceived and interpreted that could be making assumptions about intention. They may also reflect a willingness by young people to see value even in questions that make them feel ‘uncomfortable’ or are perceived as ‘negative comments’, or it is possible that adolescents’ quality of life is reflected by parental involvement in their lives and that the frequency of parental comments is a “proxy” variable for parental attention more broadly. However, these findings must be interpreted with caution, as quality of life is associated with many other aspects of people’s lives. It is possible that this is a misleading finding and would need to be replicated.

Taken together, these longitudinal results imply that there is no clear rule for whether positive or negative eating or weight/shape comments from mothers or fathers increase the risk of EDCs, BMI, psychological distress, or poor quality of life. Whilst it is salient for parents to be more aware of how they communicate within the family structure and the influence their comments have and how they are perceived, perception is not necessarily indicative of intention. Investigating causation reduces the discussion about eating disorders and parental–adolescent communication to the potential to blame parents. However, there are many contributing factors apart from parents that influence the way that comments are heard and influence quality of life, such as relationship with food, eating, weight, shape and psychological wellbeing, meaning that exclusively, parental blame may not be justified. Thus, an ideal study would look at bidirectional influences between parental commenting and disordered eating and would explore underlying intentions in comments.

One of the greatest strengths of this study includes the longitudinal design and the way in which the findings here provide an extension to our previous cross-sectional study [24]. The included cohort consisted of a mix of age and stage, which offers insight into the changing relationships between parents and adolescents as they transition through school into young adult life, and the questions were specifically designed to explore perceived weight, shape, and eating comments from mothers and fathers, from the perspective of sons and daughters. This allowed us to explore the nuances of gendered effects. However, limitations include the fact that we did not ask about recency in our survey questions or the type of family the young person belonged to, such as non-traditional families including mother/father-only households, blended families, two mothers/fathers or living with, or only living with, grandparents, etc. Furthermore, we relied on self-report, which could give rise to recall bias. Using quality of life as a measure does not account for other potential influences in adolescents’ lives that might influence our interpretation of the data. However, the psychosocial functioning subscale has shown good reliability and validity in previous studies with adolescents [43] and resulted in a valuable discussion on the perceived valence of parent comments to adolescents which adds to the existing literature [35]. A further limitation was the wide age range studied. This could be addressed in future studies by following similar age adolescents over a period of time to ensure that the adolescents’ maturity and other influencing factors particular to stages of development can be explored. Whilst the initial recruitment was a representative sample, by waves 2 and 3, there was incomplete data, which is, however, not unusual for a longitudinal study. The consequent intention to treat analysis means that the findings are not affected by attrition or loss of statistical power.

### 4.4. Public Health Implications

These findings suggest that although we cannot determine other influencing factors, the way adolescents perceive what their mothers and fathers say about weight, shape, and eating has influence over them, and that perceived positive comments do not necessarily result in better outcomes than perceived negative comments. The negative associations with these comments tend to follow similar patterns through adolescence into adult life, with sex and age playing a mediating role in the severity of those outcomes. Therefore, rather than focus on blame, it is important for health care workers, practitioners working with families, and parents themselves to be aware of the intention behind their comments and the potential influence of their communication.

## 5. Conclusions and Recommendations

The present study found that maternal and paternal comments were perceived differently by their adolescent sons and daughters. This research illustrates the complex nature of communication around weight, shape, and eating in a family environment during a key developmental period. Further studies are needed that consider the make-up of non-traditional families and consider how younger people in early adolescence interpret what is perceived as a positive or negative comment. Lastly, the findings suggest a recommendation for school and parental programs that raise awareness of how words can influence health and wellbeing, and that specific aspects may differ for mothers and fathers and their sons and daughters.

## Figures and Tables

**Table 1:** Multiple linear regression results for the effects of maternal and paternal comment variables on psychological distress, BMI, eating disorder cognitions, and paediatric quality of life at one-year follow-ups for both sexes.

DV—Psychological Distress at one-year follow-up			
Covariates	Regression Estimate	Std. Error	95% Confidence Interval
Mum Pos w/s	0.99235	0.0050978	0.67233, 1.29780 ^a^
Mum Neg w/s	0.44816	0.00702250	0.011849–0.90282 ^c^
Mum Pos eat	0.079977	0.0052465	−0.58923–0.074676
Mum Neg eat	0.236506	0.00586348	−0.43108–0.32393
Dad Pos w/s	−0.41245	0.0064572	−0.80179–−0.00603 ^c^
Dad Neg w/s	−0.23716	0.0078824	−0.73149–0.27819
Dad Pos eat	0.14059	0.0057707	−0.22183–0.47616
Dad Neg eat	0.28395	0.0066774	−0.14671–0.70540
DV—BMI percentile at one-year follow-up			
Mum Pos w/s	−0.49650	0.016336	−1.54282–0.53124
Mum Neg w/s	0.52102	0.02041	−0.72344–1.85986
Mum Pos eat	0.61500	0.010649	−0.00483–1.35082
Mum Neg eat	0.45088	0.0166	−0.60690–1.46033
Dad Pos w/s	−0.60423	0.016755	−1.65348–0.39406
Dad Neg w/s	0.28267	0.02259	−1.13324–1.65265
Dad Pos eat	−0.043025	0.016846	−1.07869–1.01132
Dad Neg eat	0.35503	0.018996	−0.78637–1.53416
DV—Weight/shape/eating concern at one-year follow-up			
Mum Pos w/s	0.071072	0.0010868	−0.024361–0.11333
Mum Neg w/s	0.042849	0.0008689	−0.055112–0.05546
Mum Pos eat	0.058965	0.000913	0.0048855–0.11281 ^b^
Mum Neg eat	0.058924	0.0002079	−0.003471–0.02198
Dad Pos w/s	−0.021558	0.00125	−0.1104–0.04778
Dad Neg w/s	−0.032013	0.000955	−0.05879–0.06186
Dad Pos eat	0.003446	0.001109	−0.03833–0.09144
Dad Neg eat	0.021666	0.000820	0.020261–0.12046 ^c^
DV—Paediatric Quality of Life at one-year follow-up			
Mum Pos w/s	0.58896	0.008383	0.030517–1.09920 ^c^
Mum Neg w/s	3.18798	0.012041	2.46844–3.93090 ^a^
Mum Pos eat	1.65036	0.009520	1.05759–2.25714 ^a^
Mum Neg eat	0.40639	0.009997	−0.21515–1.02817
Dad Pos w/s	1.99675	0.010173	1.37242–2.61878 ^a^
Dad Neg w/s	−0.91563	0.013401	−1.72149–−0.065093 ^c^
Dad Pos eat	−1.55958	0.009542	−2.12050–−0.94864 ^b^
Dad Neg eat	0.74142	0.011289	0.051909–1.43653 ^c^

Notes: DV means dependent variable; the significance levels are reported only for significant covariates. Abbreviations are as follows: w/s = weight/shape comments; eat = eating comments; all models controlled for sex and baseline stage except for the models with the DVs psychological distress and BMI percentiles at 1-year follow-up0, which controlled for baseline stage only. Significance level ^a^ = *p* < 0001, ^b^ = *p* < 0.01, ^c^ = *p* < 0.05.

**Table 2:** Multiple linear regression results for the effects of maternal and paternal comment variables on psychological distress, BMI, eating disorder cognitions, and paediatric quality of life at one-year follow-ups for daughters.

DV—Psychological Distress at one-year follow-up			
Covariates	Regression Estimate	Std. Error	95% Confidence Interval
Mum Pos w/s	0.547162	0.258327	0.04055–1.053776 ^c^
Mum Neg w/s	0.399989	0.435787	−0.47858–1.278556
Mum Pos eat	0.070161	0.358689	−0.65306–0.793381
Mum Neg eat	0.014952	0.339402	−0.66144–0.691347
Dad Pos w/s	−0.239619	0.312558	−0.85596–0.376722
Dad Neg w/s	−0.409912	0.530026	−1.48188–0.662060
Dad Pos eat	0.079544	0.339936	−0.59834–0.757428
Dad Neg eat	0.408813	0.450080	−0.50518–1.322810
DV—BMI percentile at one-year follow-up			
Mum Pos w/s	0.441931	0.952161	−1.46975–2.35361
Mum Neg w/s	−0.265757	1.077695	−2.39882–1.86731
Mum Pos eat	1.144299	0.877092	−0.58814–2.87674
Mum Neg eat	1.533354	0.992520	−0.44756–3.51426
Dad Pos w/s	−1.630189	1.051427	−3.74095–0.48057
Dad Neg w/s	0.630653	1.448449	−2.27571–3.53702
Dad Pos eat	0.205996	0.959646	−1.70052–2.11251
Dad Neg eat	0.315172	1.260646	−2.23185–2.86220
DV—Weight/shape/eating concern at one-year follow-up			
Mum Pos w/s	0.017935	0.050572	−0.08286–0.118726
Mum Neg w/s	0.058796	0.062284	−0.06462–0.182211
Mum Pos eat	−0.000387	0.063073	−0.12895–0.128173
Mum Neg eat	0.074965	0.055930	−0.03660–0.186528
Dad Pos w/s	0.015114	0.054543	−0.09326–0.123492
Dad Neg w/s	−0.154679	0.090876	−0.33977–0.030408
Dad Pos eat	−0.001000	0.061043	−0.12427–0.122268
Dad Neg eat	0.144540	0.068031	0.00784–0.281242 ^c^
DV—Paediatric Quality of Life at one-year follow-up			
Mum Pos w/s	0.136779	0.699488	−1.24697–1.52053
Mum Neg w/s	−0.594175	1.164854	−2.97719–1.78884
Mum Pos eat	−0.041412	0.912565	−1.89476–1.81193
Mum Neg eat	−0.174779	0.859763	−1.89973–1.55017
Dad Pos w/s	−0.102344	0.810269	−1.71289–1.50820
Dad Neg w/s	−0.073495	1.490394	−3.15292–3.00593
Dad Pos eat	−1.108047	0.842249	−2.79374–0.57765
Dad Neg eat	−0.609623	1.077961	−2.79703–1.57778

Notes: DV means dependent variable; the significance levels are reported only for significant covariates. Abbreviations are as follows: w/s = weight/shape comments; eat = eating comments; all models controlled for baseline stage. Significance level ^c^ = *p* < 0.05.

**Table 3:** Multiple linear regression results for the effects of maternal and paternal comment variables on psychological distress, BMI, eating disorder cognitions, and paediatric quality of life at one-year follow-ups for sons.

DV—Psychological Distress at one-year follow-up			
Covariates	Regression Estimate	Std. Error	95% Confidence Interval
Mum Pos w/s	0.449974	0.309278	−0.16153–1.061479
Mum Neg w/s	0.066467	0.432917	−0.79521–0.928142
Mum Pos eat	−0.136563	0.348827	−0.82962–0.556499
Mum Neg eat	0.279125	0.327375	−0.36798–0.926232
Dad Pos w/s	−0.493587	0.349946	−1.18727–0.200093
Dad Neg w/s	0.217218	0.527497	−0.84650–1.280934
Dad Pos eat	0.128591	0.338706	−0.54009–0.797268
Dad Neg eat	0.048717	0.373476	−0.68638–0.783817
DV—BMI percentile at one-year follow-up			
Mum Pos w/s	−1.571633	1.057317	−3.68179–0.53852
Mum Neg w/s	0.391004	1.438205	−2.49372–3.27572
Mum Pos eat	2.486129	0.976456	0.56761–4.40465 ^c^
Mum Neg eat	−0.392932	0.954827	−2.26887–1.48300
Dad Pos w/s	−0.692873	1.208114	−3.11449–1.72874
Dad Neg w/s	0.380946	1.437645	−2.46581–3.22770
Dad Pos eat	−0.676045	1.197744	−3.07228–1.72019
Dad Neg eat	0.904892	1.317012	−1.71743–3.52722
DV—Weight/shape/eating concern at one-year follow-up			
Mum Pos w/s	−0.002798	0.050791	−0.10482–0.099221
Mum Neg w/s	−0.024083	0.055280	−0.13280–0.084630
Mum Pos eat	0.008083	0.049923	−0.09103–0.107200
Mum Neg eat	0.056656	0.048117	−0.03869–0.151998
Dad Pos w/s	−0.021521	0.050928	−0.12255–0.079510
Dad Neg w/s	0.058937	0.065622	−0.07087–0.188739
Dad Pos eat	−0.000251	0.048425	−0.09577–0.095265
Dad Neg eat	−0.057333	0.057040	−0.17020–0.055538
DV—Paediatric Quality of Life at one-year follow-up			
Mum Pos w/s	−1.096001	0.872741	−2.84420–0.65220
Mum Neg w/s	0.010867	0.975776	−1.90878–1.93051
Mum Pos eat	1.866394	0.805429	0.28073–3.45205 ^c^
Mum Neg eat	−1.165935	0.892934	−2.94674–0.61487
Dad Pos w/s	1.457668	0.912779	−0.35824–3.27358
Dad Neg w/s	−1.278405	1.228301	−3.72905–1.17224
Dad Pos eat	−1.638025	0.904096	−3.43279–0.15674
Dad Neg eat	0.547419	0.969840	−1.36572–2.46055

Notes: DV means dependent variable; the significance levels are reported only for significant covariates. w/s = weight/shape comments; eat = eating comments; all models controlled for baseline stage. Significance level ^c^ = *p* < 0.05.

## Data Availability

Deidentified data are available for upon request, subject to approval from the authors’ institutional ethics committee.

## Supplementary Materials

The following supporting information can be downloaded at: https://www.mdpi.com/2072-6643/15/6/1419/s1. File S1. Quantitative questions in EveryBODY study.
