# Correction: Dahill et al. Associations between Parents’ Body Weight/Shape Comments and Disordered Eating Amongst Adolescents over Time—A Longitudinal Study. *Nutrients* 2023, *15*, 1419

**DOI:** 10.3390/nu15183993

**Published:** 2023-09-15

**Authors:** Lucy M. Dahill, Phillipa Hay, Natalie M. V. Morrison, Stephen Touyz, Deborah Mitchison, Kay Bussey, Haider Mannan

**Affiliations:** 1 Translational Health Research Institute, School of Medicine, Western Sydney University, Sydney, NSW 2051, Australiah.mannan@westernsydney.edu.au (H.M.); 2 South West Sydney Local Health District, Camden and Campbelltown Hospitals, Campbelltown, NSW 2560, Australia; 3 School of Psychology and Inside Out Institute, University of Sydney, Sydney, NSW 2006, Australia; 4 Sydney Local Health District, Camperdown, NSW 2050, Australia; 5 Centre for Emotional Health, Macquarie University, North Ryde, NSW 2109, Australia

## Text Correction

There was an error in the original publication [1]. The authors would like to correct an error in reporting the results for BMI percentile. Whilst Tables 2 and 3 are correct, the wording of the results of gendered dyads with mothers has sons and daughters the wrong way round. Mothers and sons had significant results; there were no significant results for mothers and daughters. The discussion section represents them correctly.

A correction has been made to “Section 3.2”:

There were no significant comment variables when exploring parental comments to all adolescents (see Table 1); however, when exploring gendered dyads, mothers’ positive comments to sons on eating were significantly related to higher BMI at one-year follow-up (RE: 2.49; 95% CI: 0.57–4.41 *p* < 0.05) (see Table 3). For daughters, there were no significant comment variables (see Table 2).

The authors apologize for any inconvenience caused and state that the scientific conclusions are unaffected. The original publication has also been updated.
