# Supplementary material for: Associations between Parents’ Body Weight/Shape Comments and Disordered Eating Amongst Adolescents over Time—A Longitudinal Study
Source: Nutrients. 2023 Mar 15;15(6):1419. doi: 10.3390/nu15061419 (PMC10059676; doi:10.3390/nu15061419)
Supplement: Supplementary file 1 [file nutrients-15-01419-s001.zip › nutrients-2257297-supplementary.pdf]

Additional File S1.
Quantitative questions in EveryBODY study

Is your mother in your life? (i.e., do you have regular contact with your mother) (if you have two mother figures in your life, please answer for the one you spend the most time with)

yes/no

If yes:
How frequently did your mother positively comment on your body weight/shape (e.g., “you’ve got lovely legs”)?

- Never
- Rarely
- Sometimes
- Often
- All the time

How frequently did your mother negatively comment on your body weight/shape (e.g., “you really need to lose weight”)?

- Never
- Rarely
- Sometimes
- Often
- All the time

How frequently did your mother comment positively on your eating (e.g., “Great to see you eating salad!”)?

- Never
- Rarely
- Sometimes
- Often
- All the time

How frequently did your mother comment negatively on your eating (e.g., “You shouldn’t eat so much of that”)?

- Never
- Rarely
- Sometimes
- Often
- All the time

Is your father in your life? (i.e., do you have regular contact with your father) (if you have two father figures in your life, please answer for the one you spend the most time with)

yes/no

If yes:
How frequently did your father positively comment on your body weight/shape (e.g., “you’ve got lovely legs”)?

- Never
- Rarely
- Sometimes
- Often
- All the time

How frequently did your father negatively comment on your body weight/shape (e.g., “you really need to lose weight”)?

- Never
- Rarely
- Sometimes
- Often
- All the time

How frequently did your father comment positively on your eating (e.g., “Great to see you eating salad!”)?

- Never
- Rarely
- Sometimes
- Often
- All the time

How frequently did your father comment negatively on your eating (e.g., “You shouldn’t eat so much of that”)?

- Never
- Rarely
- Sometimes
- Often
- All the time

How supported did you feel by others in your life when you received negative comments around your body weight/shape (e.g. did they support you or speak up for you)?

- Never
- Rarely
- Sometimes
- Often

- All the time

How frequently did you feel supported by others in your life when you received negative comments around your body weight/shape (e.g. did they support you or speak up for you)?

- Never
- Rarely
- Sometimes
- Often
- All the time
